# Integrating Syrian refugees into Lebanon’s healthcare system 2011–2022: a mixed-method study

**DOI:** 10.1186/s13031-024-00600-w

**Published:** 2024-05-31

**Authors:** Gladys Honein-AbouHaidar, Lama Bou-Karroum, Sarah E. Parkinson, Rima Majed, Sabine Salameh, Najla Daher, Nour Hemadi, Fouad M. Fouad, Fadi El-Jardali

**Affiliations:** 1 Hariri School of Nursing, American University of Beirut, Beirut, Lebanon; 2 Faculty of Health Sciences, American University of Beirut, Beirut, Lebanon; 3 Knowledge to Policy (K2P) Center, American University of Beirut, Beirut, Lebanon; 4 Johns Hopkins University, Washington, DC USA; 5 Department of Sociology, Anthropology & Media Studies, American University of Beirut, Beirut, Lebanon; 6 Department of Health Research Methods, Evidence, and Impact (HEI), McMaster University, Hamilton, ON Canada

**Keywords:** Refugees, Healthcare system, Health services, Lebanon

## Abstract

**Introduction:**

The Lebanese government estimates the number of Syrian refugees to be 1.5 million, representing 25% of the population. Refugee healthcare services have been integrated into the existing Lebanese health system. This study aims to describe the integration of Syrian refugee health services into the Lebanese national health system from 2011 to 2022, amid an ongoing economic crisis since 2019 and the COVID-19 pandemic.

**Methods:**

This paper employs a mixed-methods approach drawing upon different data sources including: 1- document review (policies, legislation, laws, etc.); 2- semi-structured interviews with policymakers, stakeholders, and health workers; 3- focus group discussions with patients from both host and refugee populations; and 4- health systems and care seeking indicators.

**Results:**

Although the demand for primary health care increased due to the Syrian refugee crisis, the provision of primary health care services was maintained. The infusion of international funding over time allowed primary health care centers to expand their resources to accommodate increased demand. The oversupply of physicians in Lebanon allowed the system to maintain a relatively high density of physicians even after the massive influx of refugees. The highly privatized, fragmented and expensive healthcare system has impeded Syrian refugees’ access to secondary and tertiary healthcare services. The economic crisis further exacerbated limits on access for both the host and refugee populations and caused tension between the two populations. Our findings showed that the funds are not channeled through the government, fragmentation across multiple financing sources and reliance on international funding. Common medications and vaccines were available in the public system for both refugee and host communities and were reported to be affordable. The economic crisis hindered both communities’ access to medications due to shortages and dramatic price increases.

**Conclusion:**

Integrating refugees in national health systems is essential to achieve sustainable development goals, in particular universal health coverage. Although it can strengthen the capacity of national health systems, the integration of refugees in low-resource settings can be challenging due to existing health system arrangements (e.g., heavily privatized care, curative-oriented, high out-of-pocket, fragmentation across multiple financing sources, and system vulnerability to economic shocks).

**Supplementary Information:**

The online version contains supplementary material available at 10.1186/s13031-024-00600-w.

## Background

Lebanon hosts the largest number of refugees per capita and per square kilometer in the world; by official counts, over a million Syrian and Palestinians currently reside in the country [1]. According to the United Nations High Commissioner for Refugees (UNHCR), there are 805,326 million registered Syrian refugees. The Lebanese government, however, estimates the number of Syrian refugees to be 1.5 million, which would represent for 25% of the population [2, 3].

Lebanon is not a signatory of the 1951 UN Convention Relating to the Status of Refugees or its 1967 Protocol, meaning that Lebanon cannot be a permanent country of asylum [4, 5]. In this regard, the Lebanese government considers fleeing populations as displaced persons, rather than refugees, labeling them as guests and granting them only limited, precarious legal status [6]. In contrast to neighboring countries, the state has adopted a non-encampment policy where no official refugee camps were established. As a result, Syrian refugees live within host communities, in informal settlements, and are scattered across the Lebanese territory [7]. Most refugees are concentrated in underserved, poor, and vulnerable host communities especially in North Lebanon and the Beqaa Valley as well as in crowded, impoverished urban areas [8]. They share scarce resources with host communities, served by poor public health infrastructure.

Syrian refugees dispersed within host communities have tremendous health care needs. Around 64% of the Syrian refugee population has at least one family member with a specific health need, including physical or mental disability, chronic illness, temporary illness or injury, a serious medical condition, and/or support in basic daily activities [9]. The Syrian refugee population exhibits high prevalence of chronic diseases with 46% of interviewed households reporting having at least one member with a chronic illness according to the VASyR survey in 2018 [9]. A majority of Syrians registered by UNHCR are women and children; more than half (54%) of the refugee population is under the age of 18. Of this under-18 population, 2% have a disability [9]. Family planning use is low among displaced Syrians while antenatal care needs and rates of cesarean sections are high [10]. Sexual and gender-based violence (SGBV) and mental health problems have increased in the population as a result of displacement [11].

Globally, health care for refugee populations has been provided primarily through dedicated health clinics located within refugee camps run by UNHCR or international non-governmental organizations (INGOs). As Lebanon has a non-encampment policy [12], Syrian refugees’ health services were integrated into the existing health care system as opposed to establishing parallel health services.

This study aims to describe the integration of Syrian refugees health services in the Lebanese health care system and understand its implications before 2019 and during the ongoing economic crisis using the World Health Organization (WHO) building blocks as a framework. The integration of refugees within national health systems is sometimes referred to as the “humanitarian-development nexus”. It can also refer to the extent to which host country health facilities provide equally appropriate quality services to both refugees and national populations; whether refugees health workers are able to work and provide health care services; and the financial mechanisms established to support refugee health services are integrated with financing channels employed by the host nation.

## Context

Lebanon struggled with political instability and a weak economy even before the onset of the Syrian refugee crisis, both of which hindered its capacity to meet its own citizens’ needs. A long history of civil wars, conflict, and political instability contributed to a weak of public sector and economy [13]. In addition to the state, “a range of non-state providers, including political parties, religious charities, community-based groups, NGOs, and for-profit institutions, supply and finance health care” [14]. With the onset of the Syrian refugee crisis, the ailing political and economic situation undermined the country’s ability to respond effectively to the refugee crisis; the healthcare system experienced immense strain [8, 15].

With humanitarian aid from UN agencies such as UNHCR, the WHO, and United Nations International Children’s Emergency Fund (UNICEF) as well as INGOs such as Médecins Sans Frontières (MSF), the Lebanese government has tried to partially respond to the Syrian refugees’ needs while also benefiting host communities [13]. However, as the crisis became protracted, international agencies’ aid waned, threatening sustainability. Additionally, the weakness of Lebanese political institutions, and mounting distrust towards the Lebanese government after the October 2019 uprising and the August 4, 2020 Beirut port explosion, have impacted the capacity of and the trust in the Lebanese state to manage international funding [16, 17].

Since October 2019, Lebanon has been suffering from an unprecedented economic and financial crisis, exacerbated by political instability, the COVID-19 pandemic, and the Beirut port explosion [18]. The World Bank ranked the economic and financial crisis in Lebanon among the top 10 most severe crises globally since the mid-19th century [18]. The crisis led to a 20.3% contraction in gross domestic product in 2020, an increase in poverty rates to over 50% of the population and a surge in unemployment rates [18]. The average inflation rate reached 269% in 2023 [19] with the Lebanese currency losing 98% of its value by March 2023 [20]. The economic crisis had severely impacted the health care sector, making health care unaffordable to most, leading toshortages in medicines and medical equipment, and triggering an exodus of health workers [21].

## Methodology

### Conceptual frameworks

We used the WHO health system building blocks framework (health service delivery, health workforce, health information systems, access to essential medicines, health systems financing, leadership and governance) to frame our analysis [22]. For this manuscript, we did not look at the leadership and governance; another paper focuses on integration policies.

### Study design

We employed a mixed-methods, research design triangulating four different data sources: document review, semi-structured interviews, focus group and indicator analysis.

### Document review

We examined three types of documents: (1) peer reviewed articles, (2) reports, strategies, plans and policies from governments, national and international reports and (3) news media articles. To collect peer-reviewed articles, we searched Ovid Medline, PubMed and Scopus (Appendix 1) through October 2020. To collect government, national and international agency reports, we searched websites of relevant international and government organizations (Appendix 2). Media articles were collected from the press tracing conducted by the Knowledge to Policy (K2P) Center at the American University of Beirut (AUB). K2P Press Tracing encompasses all health-related stories and issues reported in seven Lebanese and regional newspapers: anNahar, al-Akhbar, al-Hayat, al-Joumhouria, alMustaqbal, ash-Sharq al-Awsat, and The Daily Star. We only included articles on Syrian refugees located in Lebanon that were published after 2010. We focused on healthcare-related articles including access, services, delivery, systems, utilization, needs, and workers. We included papers that were not sector-specific, but when extracting data the team focused on health-relevant aspects. We excluded articles on Palestinian refugees, studies about Syrian refugees that were not based in Lebanon as well as articles about internally displaced people. We excluded articles about methodologies to conduct health research among refugees and clinical studies. Through our search, we identified a total of 284 relevant documents.

Each document retrieved was reviewed and summarized in a data collection matrix that included the title of the document, the type, the date, the actors, a summary, structural factors (political, economic, legal, historical, social, cultural), institutional factors (country’s government and health systems), individual and community factors, policy, financing of health services, availability, access and quality of health services (to host and refugees), and potential interview targets. The matrix was first piloted on a set of 20 documents where the review of documents was conducted independently by two different reviewers from the research team to achieve consensus. The results were then presented in a webinar to a number of experts from a multidisciplinary background and revised accordingly.

### Semi-structured interviews and focus groups

Interviews sought to incorporate individual participants’ views and perceptions while focus groups were meant to get the collective views and perceptions with minimal interpretation from the researchers [23].

### Data collection instruments

The team used four population-specific research instruments to collect data. First, it designed semi-structured interview protocols for three populations: (1) policymakers and decisionmakers (labelled PM, *N* = 12); (2) health care managers (labelled HML and HMS, *N* = 13), and (3) frontline healthcare providers including physicians (labelled PHL and PHS *N* = 15) and nurses (labelled RNL and RNS *N* = 17) with L and S standing for Lebanese and Syrian respectively. Interviewees were asked a mix of questions about healthcare policy and their role in its implementation as well as about their perceptions of Syrian refugee integration into the healthcare system and related policies. Second, the team deployed a focus group protocol among Syrian and Lebanese healthcare beneficiaries (labelled LB and SR, *N* = 8 focus groups for a total of 29 beneficiaries). Participants were asked to reflect on their experiences with the healthcare system over time, since the Covid-19 pandemic started, to compare their experiences of healthcare in Lebanon versus Syria (for refugees) or before 2011 versus today (for Lebanese beneficiaries), and to think through various patient scenarios and what they would advise for them. Interview and focus group protocols thus aimed to gather participants’ viewpoints and lived experiences on a range of issues using varied question formats (fact-based, opinion-based, perception-based, comparison-based, scenario-based) and to then present the resulting findings in their words.

### Sampling and recruitment

We adopted the heterogeneous purposive sampling (maximum variation) approach and the snowball technique. For policy and decision-makers, we purposefully targeted government officials such as ministers of health, heads of department, patient rights organizations such as patient advocacy groups, and international actors such as representatives from UNHCR. The research team purposefully targeted health care professionals, in regions that captured variation in integration status and refugee and host population characteristics (proximity to borders, urban, rural). In each region, we identified 2–3 health facilities (including private and public hospitals and primary health care centers) where we interviewed health facility managers and health workers including physicians and nurses. We also targeted informal refugee health workers, that is, those who are working without permission to practice but have an important role in the provision of services to refugees. Given the challenges in recruiting the latter, we adopted the snowball technique, where our formal participants were guiding us to potential informal providers of care. Beneficiaries were recruited from those attending the targeted health facilities.

We sent an email invitation to a list of potential of policy and decision makers from the different entities iterated in our sampling. In the invitation, we explained the purpose of the study, the reason they were being contacted, explained the data collection approach, and solicited their approval to participate. Those who consented were contacted by email to set a date, time and location of convenience to conduct the in-depth interviews that were hybrid (face-to-face and online) depending on the preference of the interviewees.

For health care workers, we first solicited permission from their respective administrations to approach employees. We contacted the administration of those who consented, to facilitate identifying health managers and health care workers, i.e. physicians and nurses, to coordinate with them the date, time, and location for conducting the interviews. For informal refugee health care providers, we asked the refugee providers to contact fellow colleagues who may be interested in participating in the study. Those who consented were approached.

For beneficiaries, the research team asked health facility manager to inform patients about the study including the day and the location of the focus group discussion and if they would be willing to volunteer for the study. The focus group discussion was conducted with those who those who showed up.

This study is approved by the Institutional Review Board (IRB) at the American University of Beirut (SBS-2019-0287).

### Interview and focus group implementation

After introducing themselves, the interviewers/moderators reviewed the consent form including the purpose of the study, the types of participants, and rationale for choosing them. They also emphasized the voluntary nature of participation, the option for participants to skip questions, and the right to withdraw from the study at any time without consequences. At the end, the researchers asked for permission to proceed with the questions and to record the discussion. Once approved, the audio-recording started. For those who refused to be audio-recorded, researchers took detailed notes of the discussion. Interviewers used the protocol as a data-collection guide, but asked core questions in a way that kept with the natural progression of the interview, rather than enforcing a strict order of questions. Further, they further used probing that allowed them to develop a deeper understanding of the responses and to provide more detail and nuance.

The interviewers engaged in active listening, constantly choosing the most effective approach to elicit information from the participant, while remaining neutral to any interpretation. Interviews lasted between 45 and 60 min. For the focus group discussion, the moderator played the role of the facilitator by asking questions and allowing group members to interact with each other. They intervened at times to probe for further information or to engage silent members. The focus group discussions lasted between 30 and 45 min. The interviews and focus group discussions were conducted in Arabic, the native language of participants.

### Data analysis

Audio-recordings were transcribed verbatim. The Arabic transcripts were translated in English and then validated to ensure accuracy. LBK validated the translation by randomly selecting transcripts and back translating them to ensure preserving the meaning between the original and translated data. Noteworthy to indicate that both SS and LBK share the same language and culture of participants, important to maintain the integrity of the translation process [24].

The analytical approach to the data combined both deductive framework analysis [25, 26] and inductive thematic analysis [27]. We began deductively with a set of codes based on the WHO framework, then inductively developed new codes based on themes that emerged from the data. We triangulated among the multiple data sources by assessing patterns of convergence and cross-checking themes across different data sources. The team also identified points of similarity and contrast between the different participant groups. Two data coders conducted the analysis. To protect participants’ privacy, interview and focus group transcripts were assigned anonymous codes according to the type of stakeholder interviewed (policymaker, healthcare manager, health provider, or beneficiary) and nationality (Syrian, or Lebanese). Transcripts were stored on a password-protected computer at AUB separately from the list linking the anonymous codes to participant names. Quirkos software was used for the data analysis [28].

### Indicator analysis

To complement the qualitative data, we aimed to conduct longitudinal analysis of health system and care seeking indicators. We searched for relevant health system indicators by reviewing global and regional databases of core indicators used for health systems performance. A total of 48 indicators were selected and categorized under 5 themes: health services availability (*n* = 7), health workforce (*n* = 6), health services coverage (*n* = 8), health financing and other economic indicators (*n* = 20), and health outcomes (*n* = 7). Based on the data availability and quality, we included 7 indicators in our analysis. For the indicator analysis, we set 2019 as the cutoff year for analysis to minimize the impact of confounding factors on our findings resulting from both the COVID-19 pandemic in early 2020 and the financial crisis at the end 2019, that had a negative impact on the national health information system regardless of the refugee crises.

## Results

We present the findings according to the WHO health system building blocks: health service delivery at primary, secondary, and tertiary care levels, health workforce, access to medications and vaccines, health information system, and health financing.

### Health service delivery

#### Primary health care level

The Lebanese Ministry of Public Health (MOPH) primary health care (PHC) network was a keystone in the health system response to refugees. Initially, through its Ministry-accredited network of PHCCs and dispensaries run by NGOs, the MOPH provided PHC services to the vulnerable and poor Lebanese and presented an affordable alternative to the private costly ambulatory care [29].

**Fig. 1:**
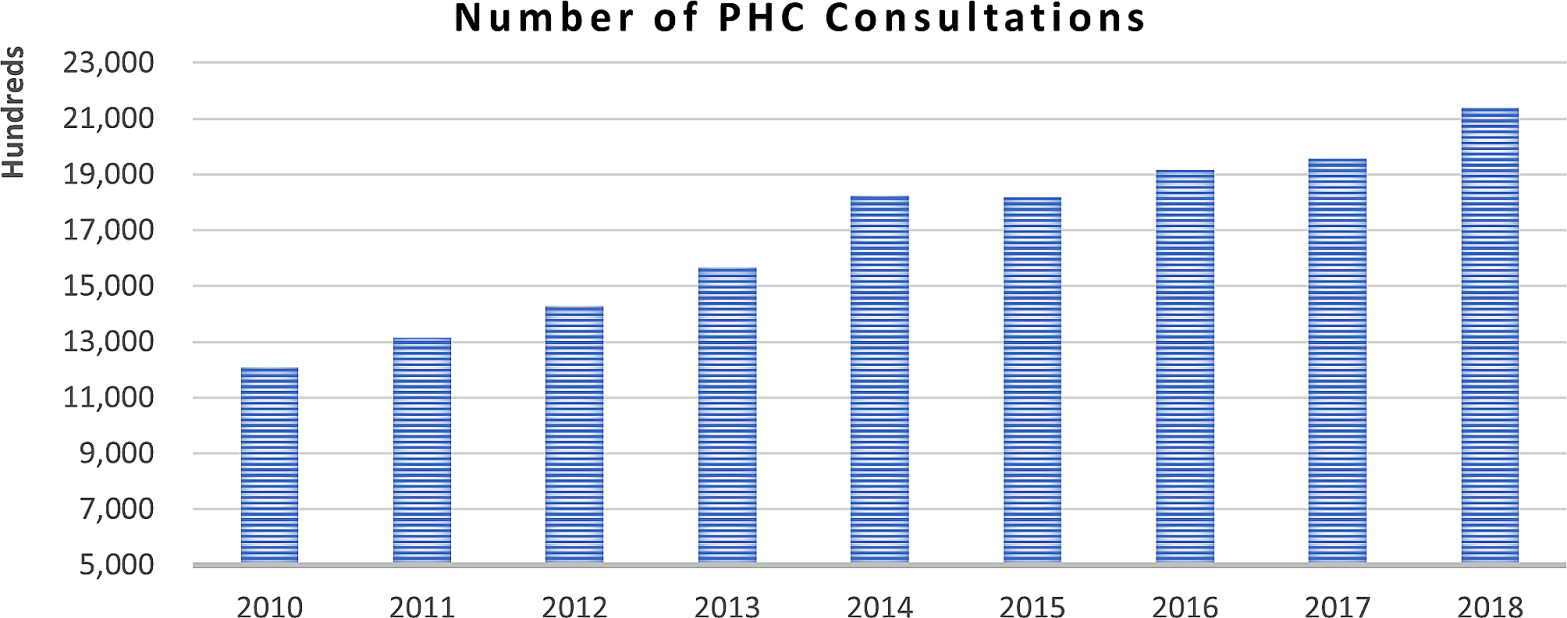
Number of PHC consultations in Lebanon including both host and refugees’ population (2010–2018) [32]

This network enabled the MOPH to provide access to displaced Syrians to PHC after the massive influx of refugees that began in 2011 [30]. Early in the crisis, the healthcare system experienced intense strain, especially in areas hosting large refugee populations such as the Beqaa Valley and Baalbek [15]. Syrian refugees were granted subsidized PHC services in PHCCs and dispensaries supported and funded by the UNHCR or its international partners [31]. According to VASyR survey in 2018, there was steady increase in demand for PHC since 2011, and access remained relatively high, with 87% of Syrian households reporting that they received the required care [9]. This dynamic was further emphasized by some of the frontline healthcare providers in our sample who stated that Syrian refugees have always had high access to PHCCs due to the low cost of subsidized services at these facilities: *“I don’t think the Syrian refugees face any challenges in accessing healthcare services… Whether registered or not at the UNHCR, … Syrian refugees are receiving the services almost free of charge, since the consultation is only 3000 L.L., in addition to benefitting from the medications available at the center” PHL-11.* This was also validated by the graph below (Fig. 1) presenting the number of PHC consultations for both the refugees and host population for the time period (2010–2018). The graph shows that the number of PHC consultations provided through the PHC network has significantly increased during the period of analysis; this number almost doubled from 1.2 million to 2.1 million visits between the years 2010 and 2018 [32].

By 2017, half of the patients in the PHCC system were Syrian [29]. According to some of our respondents, the high volume of Syrian refugee visits to PHCCs caused overcrowding, longer waiting times, and less consultation time granted to each patient which affected the quality of services provided to both communities. One health manager stated that *‘due to the overcrowding, Syrian refugees don’t get enough time during their physician consultations’ HML-11*. Some respondents from the host population expressed their frustration at long waiting times and attributed them to the large number of Syrian refugees presenting to PHCCs, which drove some to seek consultations at more costly private clinics instead: “*I arrived here at 9 am and it’s now 12:30 pm and I am still waiting for my turn, all this overcrowding is because of the Syrian refugees” LB-01.* The situation exacerbated further since the economic crisis as more Lebanese patients who had previously used the private system began visiting PHCCs, as the latter provided a cheaper and more affordable alternative to private clinics: *“I used to take my children for a consultation at a private clinic, now I always bring them to the primary health care centers…as we can’t afford to pay anymore” LB-02.*

Since the beginning of the refugee crisis, the injection of international funding allowed PHCCs to expand their resources, increase the range of medical services, and adopt better quality standards to accommodate to the increase in demand for health services. In that regard, PHCCs were, at points, able to recruit additional staff, obtain new equipment, and acquire an abundance of medical supplies. A policy-maker explained this during an interview by saying *“I believe at the higher management level of PHCCs, they consider receiving Syrian refugees as an opportunity. Because with the support they received from international donors, the primary health care centers run by international organizational and the ministry of health were able to expand their services and recruit additional staff” PM-05.* Additionally, in 2015, mental health services were integrated into PHCCs and staff members including nurses, social workers, and general practitioners, were trained accordingly [33, 34]. Reproductive and maternal health services were expanded and improved to cater to the Syrian refugees’ high need for these services, as one nurse mentioned *“Most of our maternal health beneficiaries are Syrian women so we had to recruit an OBGYN physician and get an ultrasound machine” RNL-06.* International funding also allowed for investments in accreditation as well as quality monitoring and health information systems that, according to policymakers and health providers, drastically improved the quality of care provided at PHCCs for all patients: *“The quality of the services improved drastically since the Syrian refugees’ crisis, due to the funding provided by the international organizations… it’s because of the effective support provided by international organizations that the centers were forced to work according to certain standards and to have a proper filing system. All of this didn’t exist before, it’s the international organizations’ funding that improved the quality of services” PHL-01*.

**Fig. 2:**
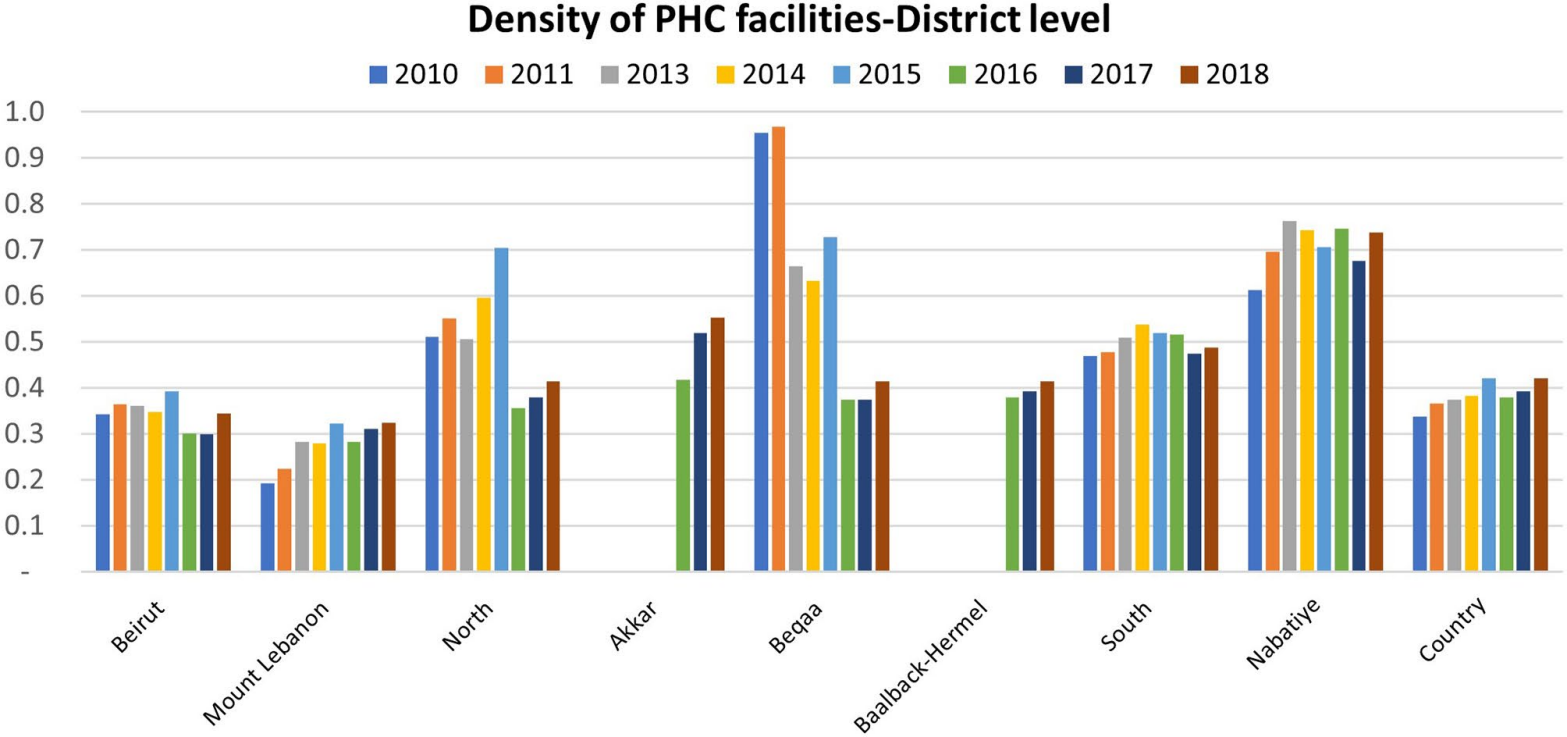
MOPH-accredited PHC facilities’ density per 10,000 population in Lebanon (2008–2019) [35–37]

Furthermore, this international funding reduced inequities in the availability of primary care services across the Lebanese territory. Many PHCCs were re-activated in remote areas that were previously neglected. New PHCCs were established to respond to increased demand. Indicator analysis validates this finding. Figure 2 presents PHCC density at the district level. The graph shows that in two remote and refugee-dense districts (Akkar and Baalback-Hermel), there were no MOPH-accredited PHC facilities prior to 2016 and that this density increased between 2016 and 2018 (from 0.4/10,000 in 2016 to 0.6/10,000 in 2018 in Akkar, and from 0.38/10,000 in 2016 to 0.41/10,000 in 2018 in Baalback-Hermel [35–37]). According to our interviewees, these new centers benefitted the host and refugee communities residing in these areas: *“I think that the Syrian crisis was an opportunity to support some additional facilities, that’s for sure. For instance, the differences between Beirut, Beqaa and South are less significant than what they were used to be in terms of availability of services”PM-01.* Policymakers and health managers also believed that providing the same services for Lebanese and Syrian refugees in the same place would decrease inequity between the two populations: “*there is equity in health services delivery since they (Syrian refugees) are receiving the service from the same place as the Lebanese” PM-07*. Figure 2 also demonstrates a noticeable decline in PHC facilities density in the North and Beqaa districts, two of the most refugee-dense districts in Lebanon. Both districts had a higher PHC facilities density rate before the refugee crisis compared to the national average. During the refugee crisis, the associated population growth shifted PHC facilities density and the ratio of patients to clinics rose, meaning that PHCC density became comparable to the national average.

#### Secondary and tertiary care

**Fig. 3:**
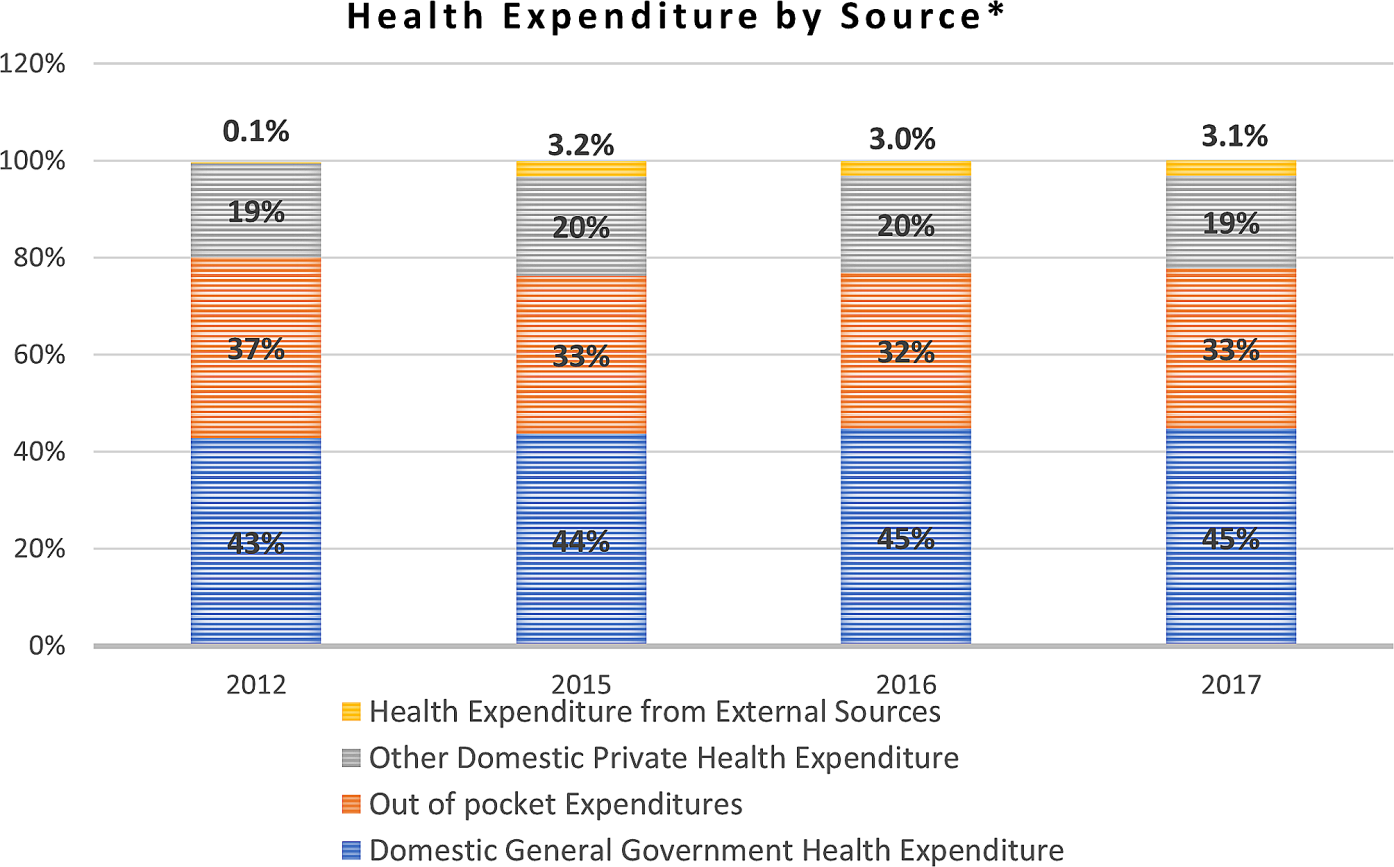
Health expenditure by source as percentage of current health expenditure [36] * *Excluding refugees*

The Lebanese health system is characterized by fragmentation due to the public–private mix involved in the financing and provision of health services [38]. Secondary and tertiary care services are mostly provided by the private sector, with 80% of hospitals owned by the private sector [39, 40]. The health system is generally curative-oriented, technology-driven, politicized, highly privatized, and characterized by high OOP expenditure accounting for 36.4% of total health spending [41]. This was validated by the indicator analysis in Fig. 3 that addressed the health expenditure by source among the host population. Although patient out-of-pocket (OOP) expenditure decreased throughout the period of analysis, the resulting 33% OOP expenditure out of total health expenditure remains high, reflecting a high reliance on patient resources and an increased financial burden shouldered by Lebanese households [36, 42, 43].

Lebanon’s history of conflict and political instability contributed to the weakening of the public health sector and the outgrowth of the private sector and local nongovernmental organizations (NGOs). The high cost of care that characterizes the highly privatized healthcare system has always impeded Syrian refugees’ access to secondary and tertiary healthcare services [44, 45]. Several respondents highlighted the challenges Syrian refugees face when accessing secondary and tertiary care, especially regarding surgeries: *“The challenge is to access hospitals, to do a surgical procedure and to do get the emergency treatment” PHS-03*. Another patient from the refugee population reported barriers to undergoing diagnostic testing: *“the challenge was also in doing the tests” SR-03.*

In this regard, several international and local NGOs, humanitarian and UN agencies, and governmental bodies were involved in the provision, subsidy, and financing of health services to provide access to Syrian refugees [46, 47]. Access to secondary and tertiary care is supported by UNHCR, which covers 75% of the cost for registered refugees (APIS, 2016). However, this assistance has in practice been limited to treatment for life–threatening and emergency conditions, with on-the-spot co-payments by refugees (OOP) many of whom could not cover the remaining 25% [48]. Along with other challenges, this reality presented a barrier to access to health services and led to heavy financial burdens on refugees. Several patient and healthcare worker respondents relayed that the main impediment to accessing secondary and tertiary healthcare services for Syrian refugees is financial:

*“The challenges the Syrian refugees face are mainly financial because the UNHCR doesn’t provide them with a full coverage of their medical expenses. For instance, many medical cases aren’t covered or are only covered partially by the UNHCR, and the out-of-pocket payment is too expensive for the Syrian refugees to bear” PHL-09.*

Further, UNHCR-subsided health care services at hospitals are only provided to registered Syrian refugees, leaving, depending on the time period, between 40% and50% of Syrian refugees who are unregistered with no formal health care coverage [45]. Our respondents emphasized that unregistered Syrians face additional hurdles when accessing hospital care: *‘At the hospital level UNHCR cover only registered refugees, the unregistered are paying out-of-pocket. In other words, you are not denying them services at the hospital level, you are not denying them the provision, and you are denying them the financing’ PM-08.* Refugees underscore diverse reasons that underly reluctance to register, including lack of information on the process, logistical challenges in reaching registration centers, fear of persecution, negative effects on their ability to return to Syria [49, 50], and the fact that the UNHCR suspended refugee registration in Lebanon in 2015 [51]. One policymaker underscored refugees’ fear of persecution: “*UNHCR registration has stopped because of the government, long time ago in 2015. Still, I believe refugees don’t like to be registered even in UNHCR; because if you’re registered it means that you’re documented, and they know where you are. Of course, in case they are registered they would be able to benefit from services and support” PM-04.*

At the start of the Syrian refugee crisis, the MOPH covered emergency care for displaced Syrians through public hospitals. Accordingly, public hospitals were an important pillar in the response to refugee crisis [44]. INGOs operated their own clinics or partnered with public hospitals to expand secondary and tertiary health services for Syrian refugees: *“Before the onset of the economic crisis, most of the Syrian refugees sought health services at public hospitals” PM-02.* After the economic crisis, the host population started accessing public hospitals more than before because of their increasing inability to afford private hospitals. One provider noted that patients lost health coverage en masse, meaning that they faced high out-of-pocket payments:

*“Before the economic crisis, there was more Syrian refugee patients presenting for care at our “public” hospital than Lebanese patients. Now, we are witnessing a surge in the Lebanese patients’ numbers in comparison to that of Syrians because we are the only public hospital in Akkar” PHL-02*.

Furthermore, after the economic crisis, hospitals started favoring Syrian refugee patients over Lebanese, which affected the equitable access between these two communities. Hospitals perceived Syrian refugee patients as more lucrative than Lebanese given that international organizations covered their hospitalization fees in US dollars. Several health managers explained that given that hospitals are facing financial challenges due to the devaluation of the Lebanese currency, care that is paid for in dollars more effectively mitigates inflation and the high operations costs they must pay to sustain operations and uphold the quality of care. One health manager stated: *“If I faced a scenario where I received three Lebanese patients and three Syrian patients and had only three vacant hospital beds. Of course, I would admit the Syrian patients. Because, unlike the Lebanese patients Syrians rely on international organizations to cover their medical expenses HML-06”.*

In fact, the economic crisis had severely affected the hospital sector, which in turn threatens access to health care for both refugees and the host population: *“The whole situation is dire and we don’t know for how long we can sustain our operations; given the ongoing economic crisis” HML-04.* The crisis has also increased tensions between the two communities due to the perceived differentiation in access to hospitals. One Lebanese health care provider stated: *“Syrian refugees are faced with a recent challenge of being resented by the Lebanese because the UNHCR health coverage made them more advantageous than Lebanese in accessing healthcare services. This resentment was not present before the economic crisis. The Lebanese do not have an appropriate health coverage (e.g. ministry of health, National Social Security Fund) like before and the hospitals prefer to serve the Syrian refugee population that is a source of fresh dollars for them. Of course, this created tension between the Lebanese and Syrian refugee population” PHL-08.*

### Health workforce

Lebanon has laws preventing non-citizens from practicing medicine in its healthcare system. Decree no.197 issued in 2014 limits possible work for Syrian nationals to the agriculture, construction and cleaning service sectors [52], a policy that mimics long-term work restrictions on Palestinian refugees in Lebanon. Despite having valuable talents that might support the overburdened Lebanese health system and public health services provided to host communities, Syrian health workers cannot practice in Lebanon due to the legal licensing and accreditation, work permits, and labor restrictions [52]. Indeed, several of our respondents directly stated that Lebanese law prevents the recruitment of Syrian heath care workers in PHCCs and hospitals, even in case of shortage:

*“The law forbids the recruitment of Syrian doctors. But the optimal thing would be for the government to legislate the recruitment of health workers from any other nationalities, to accommodate patients. Or, to resort to recruiting a Syrian job applicant when there isn’t any other Lebanese candidate available to fill the vacancy” HML-09*.

Supporters of this policy argue that competition over jobs between host and refugee populations, the risk of wage drops given an increase in labor supply, and Syrian workers’ general acceptance of lower incomes, agreement to longer work hours, and foregoing of social benefits would damage the system. Some of our Lebanese respondents stated that the Syrian refugees, in general, have taken job opportunities away from the host community, since they accept lower compensation:

*“My husband works originally in electrical maintenance but isn’t anymore because everyone prefers to hire a Syrian worker over a Lebanese one because the Syrian worker gets paid less” LB-02*.

These regulations, which ostensibly aim to protect Lebanese jobs, in fact prevent the integration of Syrian refugee health workers in the labor market [52, 53]. This dynamic has led to the emergence of informal networks of unlicensed Syrian health workers providing health services to refugees [54]. In that regard, several of our Syrian refugee health providers respondents stated that they work illicitly as nurses and physicians at PHCCs and, as a result, are not being fairly compensated. Many of our Syrian refugee physician respondents who work informally at PHCCs commented that they are losing their skills because they are unable to practice in their area of specialty. As one explains: *“I haven’t practiced surgery in 5 years now and I’m back to practicing only family medicine; which is considered a non-specialty medicine” PHS-03.*

Before the economic crisis, there was an oversupply of physicians, but a relative shortage of nurses [40]. After 2019, many highly skilled and senior healthcare providers left the country in search of better opportunities. This brain drain has hindered the quality of care and burdened the remaining staff: *“Before the crisis, we didn’t face any challenges, we had an abundance in funding and in medical staff. Now, the medical staff is emigrating and we are facing shortage in many medical specialties such as nephrology” HMS-01.*

**Fig. 4:**
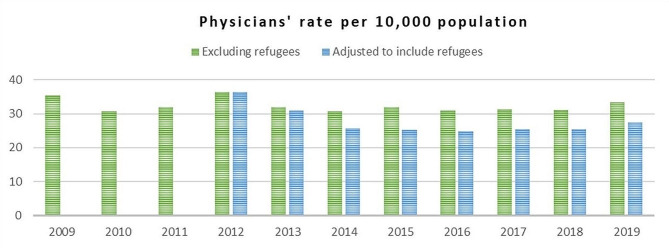
Physicians’ rate per 10,000 population in Lebanon (2009–2019) [36, 55]

Figure 4 illustrates the density of physicians (per 10,000 population) for the period preceding the economic crisis. Both figures (the adjusted and the one excluding refugees) showed a decline in the ratio of physicians per 10,000 population in Lebanon between 2012 and 2018, and then showed an increase in 2019 (from 25.4 to 27.4/10,000) [36, 55]. Despite the decline in the average number of physicians per 10,000 people, Lebanon had a much higher ratio of physicians both before and after the Syrian refugee crisis compared to other Eastern Mediterranean Region countries [56].

In the context of the economic crisis, the Lebanese health care system remains strained by the surging demand for health care in addition to the stresses of the Covid-19 pandemic. Most providers in the national system are paid in Lebanese currency, meaning that they have lost significant relative income due to currency depreciation, during a period of high work expectations and burnout. Several provider respondents voiced demands for better financial compensation: *“The main challenge is financial. As health workers, we need a greater financial compensation. This will incentivize us to provide patients with a better quality of care” PHL-08.* Informal Syrian health care workers could fill the gap in the overstretched health system. They could play a significant role in providing culturally sensitive health care to refugees and alleviate access constraints [52]. Many of our respondents argued in favor of legalizing the work of Syrian refugee healthcare providers as a solution to address provider shortages caused by brain drain related to the economic crisis. Furthermore, receiving care from health providers who are from the same nationality and culture is appreciated by the refugee community: *“It was a successful experience having Syrian physicians at our center. There are some really qualified Syrian physicians. Also, the Syrian beneficiaries felt more comfortable and at ease with physicians being from their nationality” HML-04.* Additionally, it was challenging for host health providers to deal with Syrian refugees especially at the start of the influx due to the cultural differences. One provider mentioned: *‘The difference I am witnessing is on the cultural level. The Syrian refugees coming from rural areas are less receptive than those coming from urban areas since they have lower educational level which makes it hard for us to communicate with them’ PHL-05.*

### Access to medications and vaccines

Before the onset of the economic crisis, most of the medications and basic vaccines were readily available in PHCCs for both the refugee and host communities though the MoPH [47].

**Fig. 5:**
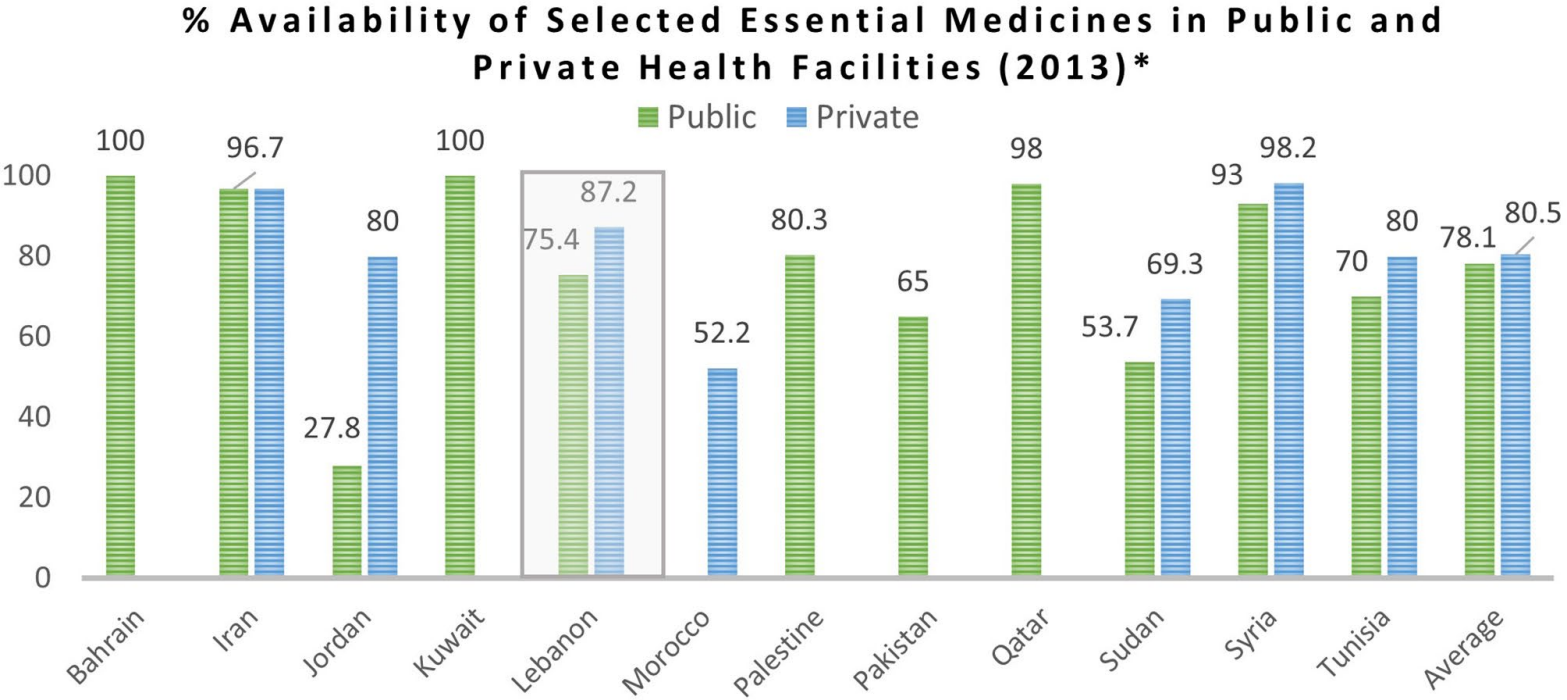
Percentage availability of selected essential medicines in public and private health facilities in the EMR countries (2013) [57]

As one patient from the Syrian refugee population stated: *“More than half of the prescribed medications used to be available at the dispensary’s pharmacy” SR-04.* Also, Syrian refugees were able to access medications in private pharmacies when needed due to their low subsidized cost: *“Before the crisis, we were able to afford buying any medication while now we can’t” RNS-01.* As shown in Fig. 5, although data concerning the availability of essential medicines in Lebanon was only reported for the year 2013, we can still conclude that during the early years of the Syrian refugee crisis the availability of essential medicines in Lebanon’s health facilities was comparable to that of other countries in the region [57].

One of the biggest challenges caused by the economic crisis, which then coincided with the COVID-19 pandemic was a countrywide medication shortage that led to the unavailability of many vital medications at private pharmacies, PHCCs, and hospitals. This medication shortage hindered access to treatment for both the host and refugee communities. Several respondents from both communities complained of their inability to find essential medications and resulting health risks. One respondent from the host community stated: *“I am a cancer patient and I have not been able to find my treatment for 2 months now. They want to kill us slowly” LB-02.* A respondent from the Syrian refugee community also reported that *“There aren’t any medications available in the dispensary’s pharmacy” SR-04.*

By 2022, many medications became available again, but host and refugee respondents reported being unable to afford their high cost at private pharmacies: *“What’s challenging is how expensive the medications are”SR-01.* The 2022 elimination of pricing subsidies on most medications led to a nation-wide price spike that further compounded barriers to access: *“Now, with the economic crisis, it became challenging for both Syrians and Lebanese to buy medications specially, when the government stopped supporting the procurement of medications” PHL-07.* Some of our refugee respondents reported that they are either rationing the use of essential and chronic medications or refraining from taking a needed prescribed medication because they could not afford it: *“Nowadays, you would tolerate the pain because you cannot afford to buy a medication. You are forced to ration when buying medications even when they are necessary” SR-04*.

In response to these issues, the MOPH made certain medications available free of charge at PHCCs for both Syrian refugees and host populations. But, according to several of our respondents, the quantity of chronic medications allocated to PHCCs was not sufficient to meet the high demand. There was consensus among all respondents about the need to increase the availability of these medications: *Our recommendation is to provide the centers with more medications especially the chronic medications that are unavailable in [private] pharmacies. Because people can’t afford to buy medications at such high prices from [private] pharmacies anymore. Those centers like ours should be more supported to continue providing services to the community” HML-05*.

**Fig. 6:**
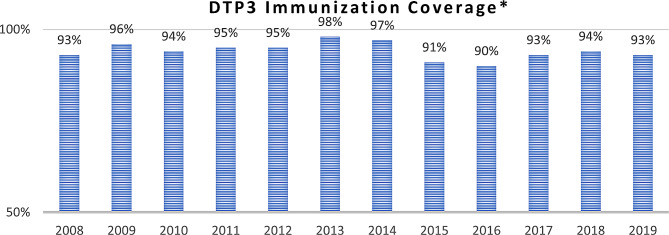
DTP3 immunization coverage in Lebanon (2008–2019) [36] * *Including refugees*

The MOPH’s Expanded Program on Immunization (EPI), as well as the massive vaccination campaigns supported by UNICEF and WHO facilitated Syrian refugees’ access to routine immunizations such as polio and measles while maintaining high vaccination rates for Lebanese [58]. In addition, the MOPH, in collaboration with UNHCR and the General Security Forces, set up vaccination centers at the borders to vaccinate Syrian children [59, 60]. Vaccines were administered free of charge at PHCCs to the host and refugee children: *“I think the main health policy in response to the refugee crisis is the provision of vaccines to all children regardless of their nationality and status” PM-05.* As shown in Fig. 6, the DTP3 immunization coverage stayed stable over the years. It was 95% in 2011, it increased gradually and reached 98% in 2013 even as the number of Syrian refugees coming to Lebanon increased during this period [36].

### Health financing

Multiple national and international organizations and governmental agencies were involved in the provision and financing of health services to Syrian refugees. For primary care, Syrian refugees benefited from subsidized services in PHCCs and dispensaries supported and funded by the UNHCR or its international partners, for a fee of 2–3 $ per consultation [47]. PHCCs are primarily managed by NGOs and part of the MOPH’s national PHC network [31]. Several respondents stated that international donors are the main source of funding for the Syrian refugees’ primary care services. UNHCR is the entity that manages the funds from foreign donors, which are then distributed through a variety of national and international NGOs. The MOPH does not directly receive any funding; instead, it works with foreign organizations to ensure that the funds are distributed through various national and international NGOs to the relevant priority areas and populations [40]. Several policymaker respondents underscored that international funding is not channeled through the MOPH due to the lack of trust in the Lebanese government and associated fear that funding might be used to serve political agendas. One policymaker stated: *“The funds are not coming to the national health system due to lack of trust in the government, funds were going to NGOS and we did not have control on the manner in which the NGOs were spending these funds. This was mostly due to political reasons” PM-07.*

The MOPH covers Syrian refugees’ **secondary and tertiary care** such as emergency services through public hospitals. Access to secondary and tertiary care was also supported by UNHCR. UNHCR covers 75% of the cost for registered refugees; refugees are responsible for a 25% OOP co-payment [44]. However, this assistance has been limited to those suffering life–threatening and emergency conditions [47, 48]:*“For the hospital care of non-COVID cases, refugees are covered mainly by UNHCR for 75% of the bill and we also have some other partners that might contribute to the remaining 25%” PM-11.*

**Fig. 7:**
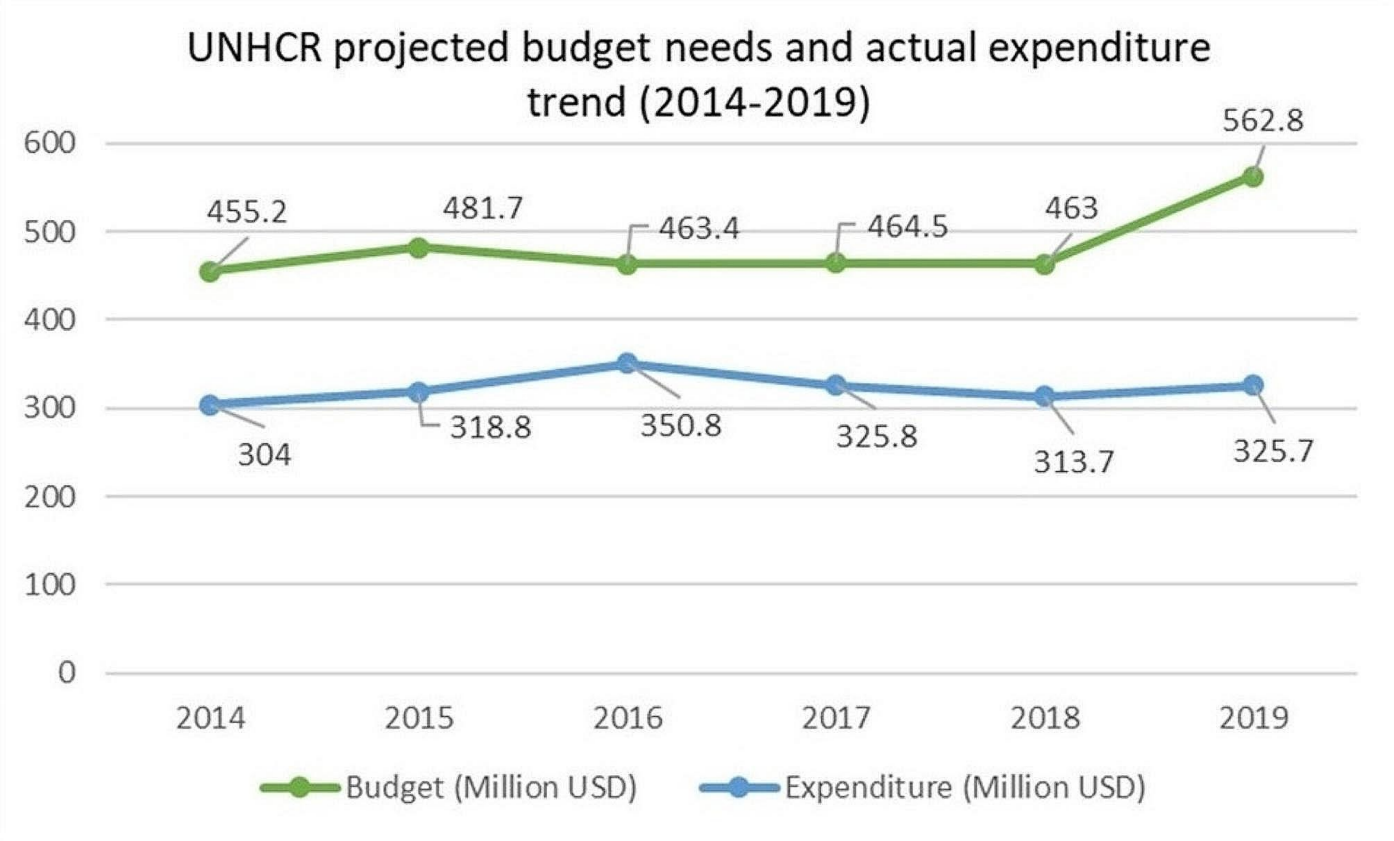
UNHCR projected budget needs and actual expenditure trend (2014–2019) [61]

Levels of international donor assistance have been inconsistent throughout the crisis. It remains considerably below the funding levels requested to provide for the refugees’ medical needs. For instance, less than 50% of the required funding was provided in 2013;this number dropped to 33% in 2014 [40]. According to several respondents, funding is decreasing with time while the health needs of the refugees and host communities are increasing, especially with the onset of the economic crisis. One policymaker stated: *“The donors are reducing their funding each year and the cost is becoming higher” PM-05*. Figure 7 represents UNHCR projected budget needs and actual expenditure trend (2014–2019). Actual financial resources spent by UNHCR Lebanon are remarkably less than the projected needs/proposed budget, with a financial gap widening from 151.2 million USD in 2014 to 237.1 million USD in 2019 (representing a 56.8% increase throughout the period of analysis) [61].

Many recommendations emerged during policymaker interviews, including for the government to play a bigger role in the Syrian refugees’ and host communities’ health response given the unsustainability and unpredictability of international funding. Several argued that the government should assume full responsibility to ensure the ongoing and uninterrupted provision of health services to the refugee and host communities: *“There has to be another mechanism that has a bigger role to the government, although the government is resisting it. I think the government should step up and play a bigger role in coordination and to have a sustainable system. For instance, there was an incidence once where the minister wasn’t going to renew the contract with the UNHCR, what would’ve happened then? We are facing the same scenario with the Palestinians, if the UNRWA funding stops what would happen then” PM-04*.

Other policymaker respondents addressed the need for strategical planning to address a protracted crisis such as the Syrian refugee influx. One highlighted the need to provide a long-term and systematic approach rather than some short-term sporadic interventions: *“There is a need to have plan for long-term solutions because the crisis in 3rd world countries are extending for a long-time period such in the case of Syria where it lasted for 11 years now” HMS-01.* Another recommendation emerged on providing sustainable financing mechanisms to Syrian refugees health services: *“We can create for them an essential health insurance plan that they can buy similar to what has been done in some African countries. This plan will have the essential health services such as immunization, and other basic services and refugees could buy for approximately $150 per year” PM-02.*

### Health information system

During the first year of the Syrian crisis, no data was collected on the number of refugees coming from Syria to Lebanon due to the unexpected nature of the crisis, historical labor migration patterns from Syria to Lebanon, and limited capabilities of both the Lebanese government and international organizations to collect such information. The UNHCR began collecting this type of data in 2012 [37]. Nevertheless, per one of the policymaker respondents, this data wasn’t reliable since not all Syrian refugees were registered with the UNHCR. This reality led to inaccuracies when calculating national health indicators: *“Can we consider the number provided by UNHCR as accurate? No, it is not, because not all Syrian refugees in Lebanon are registered with UNHCR (we have almost 900,000 reported while the actual number is around 1.4 million)” PM-08.* Indeed, research conducted in 2012 found that approximately 41% of Syrian refugees remained unregistered [8].

Moreover, health information system weaknesses impeded the provision of care to Syrian refugees. Specifically, it restricted authorities’ ability to determine the refugee population’s health needs. 2016, brought several initiatives to develop national health information systems, including the development of a MOPH health information system that linked and unified the PHC network (WHO, 2017). Also, as of 2016, the MOPH began segregating the Vital Data Observatory’s indicators (maternal mortality, neonatal mortality, live births, etc.) by nationality, which translated into better integration of the Syrian refugees at the level of the country’s Health Information system: *“By mid-2016, we started segregation for the non-Lebanese also, it was an incremental change. All of these changes were a result of the crisis, and it was because the numbers increased a lot, we couldn’t account for them. Now we know how many Syrian babies were born in Lebanon since the crisis, there are almost around 550,000 newborns in Lebanon”.* Nevertheless, this health information system is unsustainable since it highly relies on international funding which could cease at any time jeopardizing the generation of indicators and statistics.

## Discussion

This study describes the integration of Syrian refugees’ health services in the national health systems in Lebanon and how the existing health system arrangements facilitated or impeded the integration. At the service delivery level, our study highlights the integral role that the MOPH PHC network played in the refugee health response. The PHC network became the backbone of the MOPH’s Syrian refugee integration policy after the massive influx of refugees that began in 2011. Although the demand for PHC services increased over time, the system has shown resilience and provision of services was maintained and expanded. This finding aligns with another study conducted on health system resilience in Lebanon showing that PHC services were maintained and effectively expanded [40]. After the economic crisis began in 2019, the PHC centers provided a more affordable alternative to private clinics for Lebanese patients. This finding emphasizes the importance of a well-established PHC system in enabling countries to respond to refugee crisis and other public health emergencies and building resilient health systems [32, 62]. Although some respondents complained of long waiting times and overcrowding, international funding and resources enabled the expansion of primary care in terms of new services, training, and equipment. This expansion allowed the system to serve refugees while also benefiting the vulnerable host community.

On the secondary and tertiary care level, the highly privatized and expensive Lebanese health system hindered Syrian refugees’ access to services and consequently the integration of refugees’ health services. Our findings indicate that the main challenge for Syrian refugees was accessing high-cost services such as surgeries and diagnostic tests. Although public hospitals are considered weak in Lebanon and constitute only 20% of hospitals in Lebanon, they were an important pillar in the response to refugee crisis and provided an affordable alternative for the Syrian refugees. The economic crisis has caused tension between the host and refugee population due to the differentiation in access to health care as health managers are preferring to admit Syrian refugee patients as opposed to Lebanese patients. This is because Syrian refugees are covered by international organizations in US dollars at a time when the hospitals are facing financial challenges due to devaluation of the national currency.

At the level of the health workforce, the oversupply of physicians in Lebanon allowed the system to maintain a relative high ratio of physicians to population even during the refugee crisis. However, the economic crisis has caused shortages and a large exodus of physicians. Syrian health workers are not legally allowed to practice medicine in Lebanon and thus are not integrated in the Lebanese health care system. Many respondents shared recommendations to legalize the work of Syrian health workers in order to fill resulting gaps, especially in rural areas. This finding is in line with the results of another study interviewing informal Syrian healthcare workers residing in Lebanon where almost all participants recommended a policy change to enable these providers to practice formally under a temporary registration until they return to Syria [54].

Our research shows that vaccination coverage has been maintained for refugee and host populations over the years of the crisis. This was also the case with government-subsidized essential medications that were provided to refugees via PHCCs and in pharmacies. The economic crisis and end of medication subsidies severely compromised medications’ availability to refugee and host populations. At the level of health financing, our findings show that funding is fragmented across sources and is not channeled through the government. The reliance on international funding also negatively affects the sustainability of health services due to changes in funder priorities, donor fatigue and the financial capacity of the funding entity rather the actual needs of the target population. This finding corroborate another study highlighting the need for innovative and sustainable financing mechanisms to fund refugee health [63]. Lastly, the study shows the weakness of the health information system in collecting and generating data and indicators specific to refugees. A recent assessment of the situation of health data for migrants and refugees in European health information systems found that low political priority and complex governance challenges to be factors influencing the incompleteness and insufficiency of migrant and refugee data [64].

### Strengths and limitations

This study has several strengths. First, the findings of this study were based on triangulation among four different data sources (i.e. document review, interviews, focus group and indicators analysis). Second, we interviewed four different population: policymakers, health managers, health providers and beneficiaries from the host and the refugee population. This allowed to get a comprehensive understanding of the topic and capture varied perceptions. Thirdly, we targeted health providers in regions that captured variation in integration status and refugee and host population characteristics (proximity to borders, urban, rural) and by targeting private and public facilities.

One of the main limitations of this study is the inability to interview health providers in MOPH PHC network. However, to overcome this limitation, we aimed to interview PHCCs that were previously part of the network and large PHCCs that receive large numbers of refugees. Another limitation is the recall bias as the refugee crisis started in 2011 while we did the interviews in 2021–2022. We overcame this limitation through triangulating the data from interviews with document review and analysis of indicators. A final limitation is the examination of the international funding and the funding gap between requested and received funds of solely UNHCR and not the other international organization supporting the response to refugee crisis. However, this limitation might not affect the finding of funding gap as UNHCR is the main organization involved in the response.

## Conclusion

Integrating refugees in national health systems is essential to achieve sustainable development goals, in particular universal health coverage. Our findings provide some lessons learned from the case of Lebanon on integrating refugees in national health systems. The integration of Syrian refugees health services in the Lebanese health system was shown to increase the capacity of the national system through additional infrastructure, human and technical resources, training for staff, improved quality standards and expansion of services benefiting both the host and the refugee population. Pre-economic crisis, having both populations accessing same services in the same places with same quality reduced perceived inequities. However, the economic crisis has increased tensions between the two populations due to inequity in financing and access to health care.

Although, integration was shown to increase capacity of the national health system, it was challenging in such a resource-constrained and fragile setting. Challenges included the highly privatized and curative-oriented and expensive health system, the high out-of-pocket expenditures, the fragmentation across multiple financing sources, weak health information system, and system vulnerability to economic shocks. In addition, the economic crisis in Lebanon has severely impacted the ability of the Lebanese health system to secure access and availability of health services to the Lebanese and refugee populations unveiling the fragility of the health system.

Our study provides evidence to inform inclusive policies, plans, or strategies that aim to secure refugees’ healthcare access though national health system integration. First, strengthening national PHC system and public hospitals is essential to providing basic health services to refugees and vulnerable host population and responding to health threats such as Covid-19. Establishing a sustainable financial mechanism and reducing reliance on international funding are critical to maintain the integrated health services for refugees. Integrating refugee health data in national health information systems is also important in determining the refugees’ health needs and in planning and evaluating relevant health interventions and policies [64]. Exploring ways to engage refugee health workers in the health system can benefit host and refugee population especially in rural and underserved areas. As described elsewhere in this supplement, the coordination and collaboration between different actors is also an important element for successful policies on integration.

Although this study fills a knowledge gap on the barriers, facilitators and processes of the integration of health services for refugees as identified in a recent scoping review [65], comparative case studies are still needed to understand the implications of policies on integration of refugee health services in various health systems.

### Electronic supplementary material

Below is the link to the electronic supplementary material.

Supplementary Material 1

## Data Availability

Data is available upon reasonable request from the corresponding author.

## Abbreviations

INGOs: International non-governmental organizations
MOPH: Ministry of Public Health
OOP: Out-of-pocket
PHC: Primary health care
UNHCR: United Nations High Commissioner for Refugees
WHO: World Health Organization
