# Supplementary material for: Integrating Syrian refugees into Lebanon’s healthcare system 2011–2022: a mixed-method study
Source: Confl Health. 2024 May 31;18(Suppl 1):43. doi: 10.1186/s13031-024-00600-w (PMC11143570; doi:10.1186/s13031-024-00600-w)
Supplement: Supplementary file 1 — Supplementary Material 1 [file 13031_2024_600_MOESM1_ESM.docx]

**Appendix 1**

**Electronic database search**

Database: **Ovid MEDLINE(R**) and Epub Ahead of Print, In-Process & Other Non-Indexed Citations and Daily <1946 to September 12, 2019>

Search Strategy:

--------------------------------------------------------------------------------

1 refugee*.ti,ab. (9544)

2 Refugees/ (9523)

3 displaced.ti,ab. (33948)

4 Lebanon/ (3953)

5 Lebanon.ti,ab. (4067)

6 1 or 2 or 3 (45960)

7 4 or 5 (5584)

8 6 and 7 (314)

***************************

**PubMed Search strategy (September 16, 2019)**

"refugees"[MeSH Terms] OR "refugees"[All Fields] OR "refugee"[All Fields]

"Lebanon"[MeSH Terms] OR "Lebanon"[All Fields]

#1 AND #2 (366 hits)

**Scopus Search strategy (February 13, 2020)**

( TITLE-ABS-KEY ( *Lebanon* ) )  AND  ( ( TITLE-ABS-KEY ( *refugee** ) )  OR  ( TITLE-ABS-KEY ( *displaced* ) ) )  (554 hits)

**Appendix 2**

**List of agencies and organizations searched**

**Government agencies/organizations**

| Government organization | Link to website | Date searched | Number of hits retrieved | Number of documents we might include |
| --- | --- | --- | --- | --- |
| Council of ministers | <http://www.pcm.gov.lb/> | 17/12/2019 | 5 | 1 |
| Ministry of Labor | <https://www.labor.gov.lb/> | 17/12/2019 | 54 | 27 |
| Ministry of Public Health | <https://www.moph.gov.lb/> | 17/12/2019 | 61 | 29 |
| Ministry of Social Affairs | <http://www.socialaffairs.gov.lb/> | 17/12/2019 | 62 | 10 |
| Ministry of interior/ General security | <http://www.interior.gov.lb/> | 17/12/2019 | 74 | 12 |
| Ministry of economy | <https://www.economy.gov.lb/> | 17/12/2019 | 50 | 2 |
| Ministry of education | <https://www.mehe.gov.lb/> | 17/12/2019 | 63 | 11 |

**International agencies/organizations**

| International agency | Link to website | Date searched | Number of hits retrieved | Number of documents we might include |
| --- | --- | --- | --- | --- |
| Danish Refugee Council | <https://drc.ngo/> | 27/11/2019 | 7 | 2 |
| Human Rights Watch | <https://www.hrw.org/> | 27/11/2019 | 296 | 12 |
| ILO | [https://www.ilo.org](https://www.ilo.org/) | 10/12/2019 | 5 | 2 |
| IMC | <https://internationalmedicalcorps.org/> | 10/12/2019 | 78 | 25 |
| IOM | <https://www.iom.int/> | 10/12/2019 | 130 | 23 |
| International Rescue Committee – IRC | <https://www.rescue.org/> | 11/12/2019 | 298 | 16 |
| MDM | <https://www.medecinsdumonde.org/en> | 11/12/2019 | 4 | 0 |
| Mercy Corps | <https://www.mercycorps.org/> | 11/12/2019 | 87 | 11 |
| MSF | <https://www.msf.org/> | 11/12/2019 | 80 | 7 |
| Care | <https://www.care.org/> | 11/12/2019 | 59 | 7 |
| Syria Public Health Network | <http://www.syriahealthnetwork.org/> | 11/12/2019 | 1 | 0 |
| Norwegian Refugee Council | <https://www.nrc.no/> | 11/12/2019 | 80 | 15 |
| Save the Children | <https://www.savethechildren.net/> | 11/12/2019 | 14 | 4 |
| UN DESA | <https://www.un.org/development/desa/en/> | 11/12/2019 | 1 | 0 |
| UNDP | <https://www.undp.org/> | 11/12/2019 | 98 | 18 |
| UN ESCWA | <https://www.unescwa.org/> | 11/12/2019 | 40 | 5 |
| UNFPA | <https://www.unfpa.org/> | 12/12/2019 | 152 | 16 |
| UNHCR | <https://www.unhcr.org/> | 12/12/2019 | 276 | 36 |
| UNICEF | [https://www.unicef.org](https://www.unicef.org/) | 12/12/2019 | 279 | 44 |
| UN Lebanon | <http://www.un.org.lb/> | 17/12/2019 | 42 | 10 |
| UN OCHA  (relief web) | <https://www.unocha.org/> | 26/11/2019 | 74 | 44 |
| UNRWA | <https://www.unrwa.org/> | 12/12/2019 | 166 | 17 |
| WFP | <https://www.wfp.org/> | 12/12/2019 | 56 | 0 |
| WHO | <https://www.who.int/> | 12/12/2019 | 223 | 21 |
| WHO EMRO | <http://www.emro.who.int/index.html> | 12/12/2019 | 24 | 7 |
| World Bank | <https://www.worldbank.org/> | 16/12/2019 | 109 | 2 |
| Amnesty International | <https://www.amnesty.org/en/> | 9/1/2020 | 9 | 1 |

**National and regional organizations and research institutions**

| National/ regional agency | Link to website | Date searched | Number of hits retrieved | Number of documents we might include |
| --- | --- | --- | --- | --- |
| ABAAD | <https://www.abaadmena.org/> | 16/12/2019 | 38 | 8 |
| IDRAAC | [http://www.idraac.org](http://www.idraac.org/) | 16/12/2019 | 12 | 0 |
| Caritas | [https://www.caritas.org](https://www.caritas.org/) | 16/12/2019 | 49 | 1 |
| Amel association | <https://amel.org/> | 16/12/2019 | 43 | 0 |
| IFI - refugee program | <https://www.aub.edu.lb/ifi> | 16/12/2019 | 42 | 12 |
| GHI - refugee program | <https://ghi.aub.edu.lb/> | 16/12/2019 | 17 | 2 |
| Makhzoumi Foundation | <https://makhzoumi-foundation.org/> | 16/12/2019 | 8 | 6 |
| Basmeh & Zeitooneh | <https://www.basmeh-zeitooneh.org/> | 16/12/2019 | 1 | 1 |
